# Assessment of ^99m^Tc-NTP 15-5 uptake on cartilage, a new proteoglycan tracer: Study protocol for a phase I trial (CARSPECT)

**DOI:** 10.3389/fmed.2022.993151

**Published:** 2022-10-12

**Authors:** Emilie Thivat, Marion Chanchou, Sylvain Mathieu, Sophie Levesque, Tommy Billoux, Philippe Auzeloux, Nicolas Sas, Ioana Molnar, Elodie Jouberton, Jacques Rouanet, Giovanna Fois, Lydia Maigne, Marie-Josephe Galmier, Frédérique Penault-Llorca, Elisabeth Miot-Noirault, Xavier Durando, Florent Cachin

**Affiliations:** ^1^Institut National de la Santé et de la Recherche Médicale (INSERM) U1240 Imagerie Moléculaire et Stratégies Theranostiques (IMoST), Université Clermont Auvergne, Clermont-Ferrand, France; ^2^Département de Recherche Clinique, Centre Jean PERRIN, Clermont-Ferrand, France; ^3^Centre d'Investigation Clinique UMR501, Clermont-Ferrand, France; ^4^Service de Médecine Nucléaire, Centre Jean PERRIN, Clermont-Ferrand, France; ^5^Service de Rhumatologie, Centre Hospitalier Universitaire (CHU) Gabriel Montpied, Université Clermont-Auvergne, Clermont-Ferrand, France; ^6^Unité de Radiopharmacie, Centre Jean PERRIN, Clermont-Ferrand, France; ^7^Service de Physique Médicale, Centre Jean PERRIN, Clermont-Ferrand, France; ^8^Service de Dermatologie et d'Oncologie Cutanée, Centre Hospitalier Universitaire (CHU) Clermont-Ferrand, Clermont-Ferrand, France; ^9^Laboratoire de Physique de Clermont, UMR6533, Centre National de la Recherche Scientifique (CNRS)/Institut National de Physique Nucléaire et de Physique des Particules (IN2P3), Université Clermont Auvergne, Clermont-Ferrand, France; ^10^Département de Biopathologie, Centre Jean PERRIN, Clermont-Ferrand, France; ^11^Département d'oncologie Médicale, Centre Jean PERRIN, Clermont-Ferrand, France

**Keywords:** radiopharmaceutical-diagnostic use, cartilage, proteoglycan targeting, SPECT imaging, phase I

## Abstract

**Background:**

^99m^Tc-NTP 15-5 is a SPECT radiotracer targeting proteoglycans (PG), components of the cartilaginous extracellular matrix. Imaging of PGs would be useful for the early detection of cartilage disorders (osteoarthritis, arthritis and chondrosarcoma, Aromatase Inhibitor associated arthralgia (AIA) in breast cancer), and the follow-up of patients under treatment. According to preclinical study results, ^99m^Tc-NTP 15-5, is a good candidate for a specific functional molecular imaging of joints. We intend to initiate a first in-human study to confirm and quantify ^99m^Tc-NTP 15-5 uptake in healthy joints.

**Methods:**

As the clinical development of this radiotracer would be oriented toward the functional imaging of joint pathologies, we have chosen to include patients with healthy joints (unilateral osteoarthritis of the knee or breast cancer with indication of AI treatment). This phase I study will be an open-label, multicenter, dose-escalation trial of a radiopharmaceutical orientation to determine the recommended level of activity of ^99m^Tc-NTP 15-5 to obtain the best joint tracer contrasts on images, without dose limiting toxicity (DLT). The secondary objectives will include the study of the pharmacology, biodistribution (using planar whole body and SPECT-CT acquisitions), toxicity, and dosimetry of this radiotracer. The dose escalation with 3 activity levels (5, 10, and 15 MBq/kg), will be conditioned by the absence at the previous level of DLT and of a visualized tracer accumulation on more than 80% of healthy joints as observed on scintigraphy performed at ≤ 2 h post-injection.

**Discussion:**

This first in-human phase I trial will be proof-of-concept of the relevance of ^99m^Tc-NTP 15-5 as a cartilage tracer, with the determination of the optimal methodology (dose and acquisition time) to obtain the best contrast to provide a functional image of joints with SPECT-CT.

**Trial registration number:**

Clinicaltrials.gov: NCT04481230. Identifier in French National Agency for the Safety of Medicines and Health Products (ANSM): N°EudraCT 2020-000495-37.

## Introduction

Biomechanical joint function depends on cartilage integrity. For many years UMR 1240 INSERM has developed a nuclear medicine imaging strategy targeting cartilage proteoglycans *in vivo*, such as ^99m^Tc-NTP 15-5. This radiotracer is a bifunctional agent which contains in its structure a polyazamacrocycle to complex ^99m^Tc and a positively charged quaternary ammonium (QA) function for binding to glycosaminoglycans of proteoglycans, components of the cartilaginous extracellular matrix (1). This radiotracer has emerged as a promising candidate for single-photon emission computed tomography (SPECT) imaging in nuclear medicine for specific, relevant, and functional molecular imaging of cartilage (2, 3). When intravenously administered to healthy rodents or lagomorph animals, ^99m^Tc-NTP 15-5 has been observed to accumulate markedly within articular cartilage with high *in vivo* stability (4, 5). The potential of the radiotracer to bind to cartilage has also been confirmed *ex vivo* on human knee specimens (3). A selective, intense accumulation was confirmed in cartilaginous tissue. In addition, areas of deficit in tracer uptake were clearly localized in anatomical areas of altered and eroded cartilage as seen in osteoarthritis specimens (3). Numerous preclinical studies have shown its relevance in molecular imaging of cartilaginous pathologies such as osteoarthritis (2, 4), inflammatory disease (6), and chondrosarcoma (7). ^99m^Tc-NTP 15-5 nuclear imaging was recently used in the murine destabilization of medial meniscus models, to monitor *in vivo* cartilage remodeling in response to disease-modifying osteoarthritis drugs (DMOAD) (8). Diagnosis of cartilaginous biochemical alterations could enable *in vivo* studies of joint functionality (9), which cannot be evaluated by conventional imaging techniques such as CT and MRI (10, 11). In addition, these conventional imaging techniques have poor diagnostic performances in early-stage osteoarthritis, which is a real public health problem (12–15). Functional articular cartilage imaging is of interest in joint treatment-related toxicities, such as Aromatase Inhibitor-Arthralgia (AIA). Aromatase inhibitors (AIs) are standard care for the adjuvant treatment of early hormone-sensitive breast cancer. However from 20% to 50% of women experience joint pain under AI treatment, with up to 20% treatment discontinuation for this toxicity which can compromise the effectiveness of these therapies (16, 17).

As the clinical development of this radiotracer would be oriented toward the functional imaging of cartilage in degenerative cartilage pathologies, we intend to carry out this first clinical proof-of-concept study on this radiotracer among patients (osteoarthritis and breast cancer) rather than among healthy volunteers. The evaluation of this radiotracer in this population (patients with unilateral osteoarthritis or patients with breast cancer with an indication for adjuvant AI treatment) will make it possible to verify: i) its binding to healthy joints, and ii) specific conditions enabling an initial evaluation of osteoarthritic joints.

This CARSPECT trial is thus a first in-human clinical study designed to confirm tracer accumulation in healthy cartilage joints. The main objective is to determine the recommended dose (activity in MBq/kg) of ^99m^Tc-NTP 15-5 to optimize joint fixation contrast on scintigraphy acquisitions, avoiding any toxicity. The secondary objectives relate to pharmacology, biodistribution, toxicity, and dosimetry evaluation. Differences in tracer accumulation between healthy and degenerative joints will also be assessed visually and quantitatively.

## Methods and analysis

### Study design

This “first in-human” phase I study is an open-label, multicenter, dose-escalation trial on a radiopharmaceutical tracer. The main objective is to identify, among three levels of ^99m^Tc-NTP 15-5 activity, the *recommended* (accurate) dose to detect tracer accumulation in a sufficient number of healthy joints, avoiding any toxicity.

**Figure 1:**
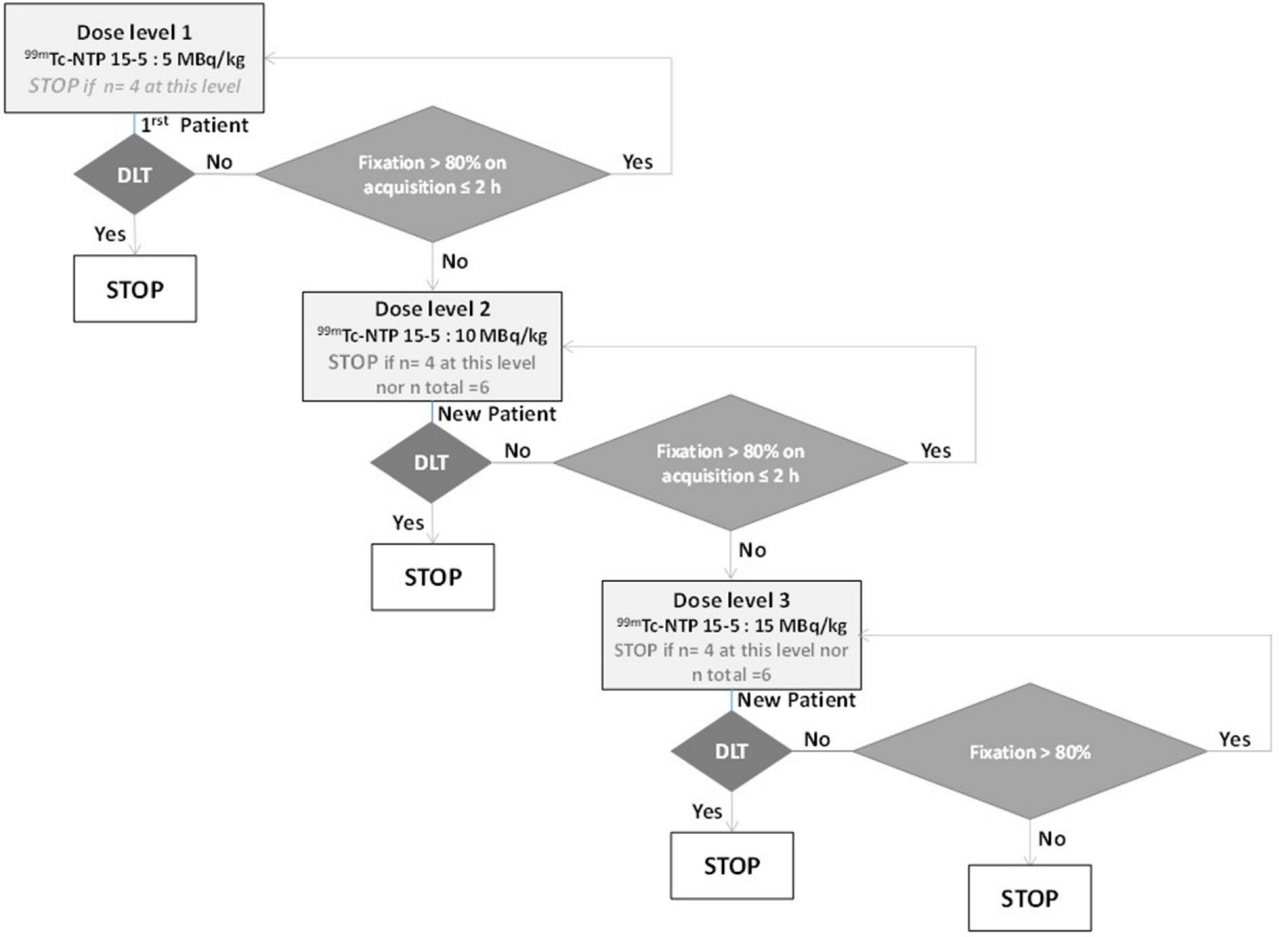
Dose escalation plan of CARSPECT study.

Dose escalation will include 3 activity levels: 5, 10, and 15 MBq/kg. The initial dose of 5 MBq/kg will be used as the first level. The dose escalation scheme will allow dose escalations of one level conditioned by the absence, at the previous level, of dose-limiting toxicity (DLT) and the presence of tracer accumulation in healthy joints appearing on scintigraphy performed at ≤ 2 h post-injection (Figure 1). With a semi-quantitative visual scale, a score of 3 for at least 80% of healthy joints was chosen because it corresponds to a sensitivity of 80%, considered sufficient for diagnostic imaging.

A maximum of 6 patients will be included in the study. A maximum of 4 patients will be included per dose level. The rule for termination will be that at least 2 patients have received the recommended dose level, with one patient from each group in the selected dose level. Recruitment will be carried out alternately in each of the 2 groups (breast cancer patients/osteoarthritis patients) as far as possible.

The recruitment period will be 24 months, with a follow-up period of 1 week, for a total duration of 25 months.

### Coordination and participating institutions

The sponsor and body responsible for the coordination of the trial, data management, monitoring, and statistical analyses will be the Center Jean Perrin, Clermont-Ferrand.

The multicenter study is currently based at 2 sites in France: The University Hospital of Clermont-Ferrand and Center Jean Perrin. Patients with osteoarthritis will be recruited from the Rheumatology Department of the University Hospital of Clermont-Ferrand and patients with breast cancer will be recruited at the Jean Perrin Cancer Center. For all patients, radiopharmaceutical injections will be performed at the Nuclear Medicine Department of the Jean Perrin Cancer Center.

### Study objectives and endpoints

#### Primary objective and endpoint

The objective of this phase I trial is to determine the recommended dose (activity in MBq/kg) of ^99m^Tc-NTP 15-5 corresponding to an optimal joint tracer contrast, analyzed on a semi-quantitative visual scale.

The tracer accumulation observed (whole body imaging) on healthy joints (according to clinical evaluation by rheumatologist) (*n* = 31) of the wrists, elbows, shoulders, lumbar, and spine joints, C6-C7, hips, knees, and ankles will be scored on a semi-quantitative visual scale using three levels:

1. Lower level of fixation than the adjacent diaphyseal uptake,
2. Fixation level equal to the adjacent diaphyseal uptake,
3. Fixation level higher than the adjacent diaphyseal uptake.

The visual analysis will be performed by 2 nuclear physicians with reconciliation of the results. In case of disagreement, consensus will be reached *via* a joint reassessment.

For each patient, the ratio of the number of healthy joints scored at level 3 to the total number of healthy joints studied will be calculated.

The recommended dose will be defined as the dose at which 100% of patients present a score of 3 in at least 80% of healthy joints as seen on scintigraphy performed at 2 h post-injection or less, and without DLT. DLT is defined as any grade 3–4 toxicity occurring in the week following the injection.

#### Secondary objectives and endpoints

The secondary objectives and the corresponding endpoints are:

- Analysis of the impact of activity level and acquisition time on tracer accumulation assessed by visual and quantitative analysis. A 3D quantification will complete the semi-quantitative visual analysis. SPECT Volumetric Regions of Interest will be centered on 7 cartilaginous zones (three lumbar discs, two hips, two knees) then on the corresponding bony diaphyses or vertebral body or the adjacent muscles. The articular uptake of ^99m^Tc-NTP 15-5 will thus be normalized to bone or muscle uptake.
- Analysis of pharmacokinetics (including excretion), biodistribution, and dosimetry of ^99m^Tc-NTP 15-5; the evolution of tracer activity/mL (AUC, Tmax and Cmax) will also be measured on whole blood and on plasma in the first 8 hours. Tracer urinary elimination over 8 h will be measured by counting.

Whole body planar (2D) and SPECT (3D) acquisitions will be performed for bio-distribution analysis and quantification of absorbed doses of tracer in target and non-target organs using a Monte-Carlo simulation and MIRD formalism (18). The analysis of ^99m^Tc-NTP 15-5 uptake intensity between osteoarthritis joints and normal joints will be carried out using visual methods and quantitative 3D analysis.

- The tolerance of ^99m^Tc-NTP 15-5 will be evaluated according to NCI-CTCAE (version 4.03). Tolerance assessments will include clinical examination, recording vital signs (heart rate, blood pressure, temperature, respiratory rate), weight, height, and OMS performance status; electrocardiogram (ECG); blood tests including full blood counts, prothrombin time, sodium, potassium, chloride, calcium, phosphorus, bicarbonate, serum total protein, serum albumin, fasting blood glucose levels, Thyroid-stimulating hormone (TSH), free thyroxine (fT4), free triiodothyronine (fT3), creatinine, urea, Alkaline phosphatase (ALP), Gamma-glutamyl transferase (GGT), Aspartate amino transferase (AST), Alanine amino transferase (ALT); 24-h urine collection tests: glucose, urea, creatinine, albumin, sodium, potassium, magnesium, calcium, and protein electrophoresis. Reporting of serious adverse events and suspected unexpected serious adverse reactions will be carried out according to the local regulations.

### Patients

#### Inclusion criteria

Inclusion criteria specific to group 1:

- Patients with painful unilateral osteoarthritis of the knee, such as femorotibial pattern defined by a radiographic score of 0/1 (Kellgren/Lawrence), and an average WOMAC score of 4 or more and by minor disorders on MRI (MOCART 2.0 score > 70).

Inclusion criteria specific to group 2:

- Patients with non-metastatic breast cancer, hormone receptor-positive, HER2-negative, with indication for adjuvant therapy with AI; treatment not yet started.
- Age < 60 years.

Common inclusion criteria:

- Patients with at least 31 healthy joints (based on clinical assessment).
- Signed written informed consent.
- Affiliation to a health insurance plan.
- For women of childbearing age (fertile, from menarche and to post-menopause unless sterile subsequent to surgery) including patients under GnRH agonist for ovarian suppression: negative serum pregnancy test at inclusion (fewer than 7 days before injection of ^99m^Tc-NTP 15-5). Menopause is defined as amenorrhea for at least 12 consecutive months without other cause and a level of FSH and estradiol consistent with levels of the post-menopausal period.
- Willing and able to comply with study visits, treatment, examinations, and the protocol.

#### Non-inclusion criteria

- Patients < 18 years of age.
- Pregnant or lactating patient.
-BMI > 30.
- History of known allergy to excipients present in the solution of ^99m^Tc-NTP 15-5.
- Chronic inflammatory rheumatism (rheumatoid arthritis, spondyloarthropathy, psoriatic arthritis, etc.) diffuse arthritis (at least 3 joints affected), autoimmune connectivitis, fibromyalgia.
- Known chronic joint pathology: osteoarthritis affecting at least 3 joints, autoimmune disease, inflammatory rheumatism (except unilateral knee arthritis).
- Individuals deprived of their freedom, under guardianship/curatorship, or under legal protection measures.
- Treatment with NSAIDs or cessation under 48 h previously.
- Inability to comply with medical requirements/follow-up of the trial for geographic, family, social, or psychological reasons. These conditions should be discussed with the patient before registration in the study.

### Intervention

#### Study drug and administration

^99m^Tc-NTP 15-5 is synthesized by radioactive labeling with ^99m^Tc of the chemical precursor NTP 15-5, N-[(3-triethylammonio)propyl]- 1,4,7,10,13-pentaazacyclopentadecane, in presence of sodium pertechnetate (19). The synthesis of the pharmaceutical grade NTP 15-5 cold precursor is manufactured in accordance with GMP (Eras Laboratoire). The cold kit is manufactured in the experimental radiopharmacy unit in the Jean Perrin Center, in the form of a single-dose sterile kit.

The experimental drug is an injectable solution of ^99m^Tc-NTP 15-5with a molar activity greater than 2.5 GBq/μmol and radiochemical purity required to be over 90%.

According to the dose escalation plan, a single injection of 5, or 10, or 15 MBq/kg of ^99m^Tc-NTP 15-5 will be administered by slow intravenous injection on D0 in the presence of a nuclear medicine physician, without any premedication. Any treatment with NSAIDs will be discontinued at least 48 h before injection and can be resumed 24 h after injection. Prior and concomitant treatments will be collected at baseline and throughout the study.

#### Study procedures and participant timeline

Before any study-related assessment starts, written informed consent will be obtained from each patient. At screening/baseline (within 15 days before administration of ^99m^Tc-NTP 15-5), the patients will have a clinical evaluation noting vital signs, ECG, a specialized consultation with a rheumatologist, blood and urine tests, and a pregnancy test (for women of childbearing age), and for patients with osteoarthritis, an evaluation of osteoarthritis by MRI and X-ray dating from less than 2 months prior.

After checking the eligibility criteria, a dose allocation request will be performed using an eCRF.

The ^99m^Tc-NTP 15-5 dose at an activity level of 5, or 10, or 15 MBq/kg (according to the dose escalation plan), will be injected on D0 with a follow-up of vital signs: before, and 5 min, 10 min, 15 min, 30 min, 1 h, 2 h, 4 h, and 6–8 h after the end of infusion.

*e In vivo* biodistribution of ^99m^Tc-NTP 15-5 will be assessed using:

- Whole-body planar scintigraphy acquisition 30 min, 2 h, 4 h, and 6–8 h after injection on D0; SPECT acquisitions at 1 h, 2.5 h, 4.5 h, and 6–8 h after injection on D0; Computed Tomography will be carried out before the injection or at the time of SPECT acquisition.
- Blood samples for the pharmacokinetic study will be collected at 5 min, 10 min, 15 min, 30 min, 1 h, 2 h, 4 h, and 6–8 h post injection on D0. An 8-h urine collection will be performed for excretion evaluation.

Patients will be followed for 1 week with a visit on D8 for safety evaluation with clinical examination, vital signs, blood and urine analyses, and ECG.

### Statistical considerations

A maximum of 6 patients should be included.

The number of subjects to be included takes into account the population selected and makes it possible to validate the hypothesis that healthy cartilage will present a significantly higher fixation than the adjacent diaphyseal fixation. For each patient, 31 observations (healthy joints) at 4 different times will be available for the evaluation of the main objective.

The optimal level of activity will be chosen using the dose escalation algorithm (Figure 1). The recommended dose will be defined as the dose from which 100% of patients present a score of 3 in at least 80% of healthy joints as observed on scintigraphy performed 2 h post-injection or less, and without DLT. If more than one time period (30 min or 2 h) meets these conditions, to choose the optimal time, we will perform a McNemar test where the number (minimum 2 patients, providing *n* ≥ 62 healthy joints) provides a power of 0.8, with an average effect size of 0.3 and an alpha risk of 0.05. For the moment, we do not have any element enabling estimation of the correlation with each patient.

Due to the nature and the design of the study, statistical analyses will be mainly descriptive. Missing data will not be replaced. The statistical significance threshold is set at 5%. Statistical analyses will be performed using R software.

### Data management and monitoring

An eCRF based on the Web-Based Data Capture (WBDC) system “Ennov Clinical” will be used for data collection, data management, and monitoring. Health-related personal data captured in the course of this study is strictly confidential and accessible only by investigators and authorized personnel. The investigator ensures the accuracy, completeness, and relevance of the data recorded (pseudonymized patient data) and of the provision of answers to data queries.

Compliance with the study protocol and the procedures therein, and the quality of the data collected (accuracy, missing data, consistency with the source documents) will be regularly reviewed by on-site monitoring and central data monitoring. Monitoring reports will ensure traceability.

### Independent data monitoring committee

The IDMC will review all safety problems or other issues identified during the medical review and seek advice as needed. Experts in the IDMC performing this review will be selected for their relevant clinical trial/medical expertise. The committee will include a nuclear physician, a rheumatologist, and an oncologist. Given the very small number of DLTs expected, the committee will meet for each severe toxicity observed (grade ≥ 3).

### Trial status

The CARSPECT trial is currently recruiting. Participant recruitment began in November 2020 with a 24-month enrollment period and an estimated completion in December 2022. The approved protocol is version 5, 15 March 2022.

## Discussion

The CARSPECT trial is the first in-human evaluation of ^99m^Tc-NTP 15-5 as a cartilage imaging radiotracer. This proof-of-concept study aims to confirm ^99m^Tc-NTP 15-5 uptake in healthy joints and to determine the recommended dose (activity in MBq/kg) of ^99m^Tc-NTP 15-5 to obtain the best joint fixation contrast on scintigraphy acquisitions, avoiding toxicity.

As observed in preclinical studies, ^99m^Tc-NTP 15-5 uptake in healthy joints is expected to be intense, with high contrast compared to adjacent diaphyseal uptake or adjacent vertebral body. Our hypothesis is that at least 80% of the healthy joints of a patient will significantly bind ^99m^Tc-NTP 15-5 (taking into account the resolution limits of the cameras).

Once this phase I trial is completed and the optimal injected activity of ^99m^Tc-NTP 15-5 validated, phase II clinical trial will be designed to address clinical questions in both degenerative/inflammatory and tumoral diseases. Clinical development of a functional articular cartilage imaging radiotracer such as ^99m^Tc-NTP 15-5 should be of interest in presence of pathological joints: osteoarthritis, arthritis, and chondrosarcoma, and in clinical situations where drugs induce severe articular cartilage toxicity, as with AI treatment in breast cancer.

Functional imaging such as proteoglycan tracer scintigraphy could be used to identify patient subgroups with PG altered cartilage, for which joint prosthesis is expected to really improve articular function and mobility. A recent preclinical study in a murine destabilization of medial meniscus model, highlights the potential of ^99m^Tc-NTP 15-5 as an imaging-based companion to monitor cartilage remodeling in osteoarthritis and response to the disease-modifying osteoarthritis drug, sprifermin (rhFGF-18), one of the most promising anabolic agents that have demonstrated cartilage repair properties in patients (8).

Further to this, in hormone-responsive early breast cancer, arthralgia has been reported for up to 50% of women treated with aromatase inhibitors, and this affects compliance and thus the response to treatment (16, 17). Functional imaging using ^99m^Tc-NTP 15-5 could provide a better understanding of the pathogenic mechanisms of arthralgia under AI and could identify patients at risk and improve their management.

Given the affinity of QA for cartilage, QA derivatives offer the possibility of an approach targeting cartilage in imaging, and also for therapies in joint diseases, serving as carriers able to selectively deliver drugs. Preclinical studies have demonstrated the possibility of using QA as vector for D-glucosamine, and DMOAD (20), or anti-inflammatory drugs (21) in arthritis treatment. Another targeted therapy developed for chondrosarcoma uses QA as a targeting strategy by addressing selectively to chondrosarcoma cytotoxic drugs such as melphalan, an alkylating agent (7). An interesting application of ^99m^Tc-NTP 15-5 imaging is as a “methodology companion” for innovative therapeutic approaches to degenerative and tumoral pathologies of the cartilage, using a proteoglycan targeting strategy.

## Ethics statement

The studies involving human participants were reviewed and approved by Comité de Protection des Personnes Sud-Ouest et Outre-Mer II. The patients/participants provided their written informed consent to participate in this study.

## Author contributions

Conception and design: FC, ET, and IM. Investigators of the study: FC, MC, SM, and XD. Review of study design and protocol: SL, PA, TB, NS, GF, LM, EJ, IM, ET, FC, JR, XD, FP-L, and EM-N. Study coordination: FC and ET. Data management and statistical analysis: IM. Obtaining funding and supervision: FC and EM-N. Drafting ^99m^Tc-NTP 15-5 IMPD: PA, M-JG, SL, EM-N, and FC. Drafting the manuscript: ET and MC. All authors contributed to revision, adaptation, and final approval of the manuscript and accountable for all aspects of the work.

## Funding

The trial will be funded by the French government IDEX-ISITE initiative 16-IDEX-0001-CAP 20-25 (Challenge 3: mobility-2017) of the University of Clermont Auvergne. The toxicology regulatory studies needed for the IMPD of 99mTc-NTP 15-5 were supported by the French National Research Agency (ANR 15-CE18-003). The funding parties were not involved in the design or conduct of the study, nor in the collection, management, analysis, and interpretation of the data. They were not involved in the writing of the manuscript.

## Conflict of interest

The authors declare that the research was conducted in the absence of any commercial or financial relationships that could be construed as a potential conflict of interest.

## Publisher's note

All claims expressed in this article are solely those of the authors and do not necessarily represent those of their affiliated organizations, or those of the publisher, the editors and the reviewers. Any product that may be evaluated in this article, or claim that may be made by its manufacturer, is not guaranteed or endorsed by the publisher.

## Abbreviations

AD: absorbed dose
AIA: Aromatase inhibitor arthralgia
AI: Aromatase inhibitors
ALP: Alkaline phosphatase
ALT: Alanine aminotransferase
ANSM: French Agency for the Safety of Medicines and Health Products
AST: Aspartate aminotransferase
CTCAE: Common Terminology Criteria for Adverse Events
DMOAD: disease-modifying osteoarthritis drugs
DLT: dose-limiting toxicity
ECG: Electrocardiogram
fT3: free triiodothyronine
fT4: free thyroxine
GGT: Gamma-glutamyl transferase
IDMC: Independent Data Monitoring Committee
MIRD: Medical Internal Radiation Dose Committee
NSAIDs: Non-steroidal anti-inflammatory drugs
PG: Proteoglycans
QA: quaternary ammonium
SPECT-CT: Single Photon Emission Computed Tomography/Computed Tomography
TRT: Targeted radionuclide therapy
TSH: Thyroid-stimulating hormone
WBDC: Web-Based Data Capture.
